# Importance of Appropriate and Substantial Imaging and Pathological Information for Rare Conditions

**DOI:** 10.5704/MOJ.2503.018

**Published:** 2025-03

**Authors:** J Ichikawa, T Kawasaki, K Onohara

**Affiliations:** 1 Department of Orthopaedics, University of Yamanashi, Chuo, Japan; 2 Department of Pathology, Saitama Medical University International Medical Center, Hidaka, Japan; 3 Department of Radiology, University of Yamanashi, Chuo, Japan

Dear editor,

We read with interest the paper by Indra *et al* titled “Pigmented Villonodular Synovitis (PVNS) of the Knee mimicking Septic Arthritis in a Paediatric Patient: A Case Report”^1^. We would like to thank the authors for publishing this interesting case. Our comment highlights the imaging and pathological findings that are required to better understand the nature of this case. It has been reported that PVNS (currently unified in tenosynovial giant cell tumour) is sometimes difficult to distinguish from juvenile idiopathic arthritis, haemophilic arthropathy, and other malignant disorders in paediatric cases^2^. Magnetic resonance imaging (MRI) plays a pivotal role in differential diagnosis. Determining the extent of inflammation and the presence or absence of tumours on a single MRI image is extremely challenging. If other cross-sectional information were available, such as FS or STIR on T2WI or contrast-enhanced T1WI, it would be easier for the reader to determine the extent of inflammation (not only intra-articular but also bone marrow) and differentiate it from tumours. T2*-WI is also useful for detecting hemosiderin.

Regarding pathological findings, the morphological characteristics of PVNS include various proportions of mononuclear cells and multinucleated giant cells, foamy macrophages, inflammatory cells, hemosiderin, and collagenisation of the stroma^3^. In this case, we could not clearly recognise mononuclear cells or multinucleated giant cells, which are observed in typical PVNS. Therefore, differentiation from hemosiderotic synovitis is challenging. In recent years, the usefulness of clusterin and colony-stimulating factor 1 in PVNS using immunohistochemistry (IHC) has been reported^4,5^, suggesting that the diagnosis of PVNS will be more robust by presenting characteristic morphological findings and further performing IHC.

In conclusion, atypical and difficult-to-diagnose cases are clinically valuable and provide new medical knowledge. However, appropriate and sufficient information must be provided to enhance the value of rare cases.
