# Supplementary material for: Importance of Appropriate and Substantial Imaging and Pathological Information for Rare Conditions
Source: Malays Orthop J. 2025 Mar;19(1):131–2. doi: 10.5704/MOJ.2503.018 (PMC12022715; doi:10.5704/MOJ.2503.018)
Supplement: Related texts

# Authors’ Reply to the Letters to the Editor

**Published:** 2025-03

**Authors:** Faris Indra, Mohd Anuar Ramdhan Ibrahim, Elsonmond Vick Duin, Dayangku Norlida Awang Ojep

**Affiliations:** 1 Department of Orthopaedics, Universiti Malaysia Sarawak, Kota Samarahan, Malaysia; 2 Department of Orthopaedics, Universiti Malaysia Sabah, Kota Kinabalu, Malaysia; 3 Department of Pathology, Universiti Malaysia Sarawak, Kota Samarahan, Malaysia

It is true that the clinical manifestation of knee pigmented villonodular synovitis (PVNS) might resemble that of septic arthritis^1,2^. Joint swelling, pain, and reduced range of motion are symptoms of both conditions that may sometimes render diagnosis challenge^1,2^.

PVNS is generally a non-infectious condition, often seen in younger individuals, while septic arthritis occurs in any age group with risk factors (e.g., recent joint surgery, immunocompromised status^3^. In septic arthritis, joint fluid analysis typically shows a high white blood cell count, with a predominance of neutrophils^1-3^. In PVNS, the fluid may be bloody or have a cell count that is elevated but not as extreme as in septic arthritis^1,2^. MRI and ultrasound can help differentiate the two^1,2^. PVNS may show characteristic nodular masses and synovial thickening, while septic arthritis might present with more diffuse joint effusion and bone marrow edema^1-3^. PVNS is usually treated with surgical intervention (e.g., synovectomy), whereas septic arthritis requires drainage and antibiotics^1-3^.

If there's suspicion of either condition, prompt evaluation and investigation are crucial^4^. In our practice any suspicion would require MRI for correct diagnosis. We usually perform tissue biopsy first for diagnosis before definitive surgery is being performed. Total synovectomy is the treatment of choice performed arthroscopically or through open surgery^4,5^.

## References

[1] Marshall AS, Molina AL, Reiff D, Sheets R, Conklin M (2020). Pigmented Villonodular Synovitis Mimicking an Acute Septic Hip in an Eight-Year-Old Male.. Cureus..

[2] Honig E, Harris A, Sabharwal S, Levin A, Honcharuk E (2022). Tenosynovial Giant Cell Tumor Mimicking Acute Septic Arthritis of the Hip: A Case Report.. J Am Acad Orthop Surg Glob Res Rev..

[3] Wang J, Wang L (2021). Novel therapeutic interventions towards improved management of septic arthritis.. BMC Musculoskelet Disord..

[4] Song HQ, Wu GF, Qi WZ, Lin LJ (2023). Diffuse pigmented villonodular synovitis treated with arthroscopic total synovial peel.. BMC Surg..

[5] Giai Via R, Giachino M, Elzeiny A, Santarsiero G, Cipolla A, Pantè S (2024). Arthroscopic Management of Pigmented Villonodular Synovitis of the Hip: A Systematic Review.. J Clin Med..
